# Acute Kidney Injury in Patients with Acute Myocardial Infarction Undergoing Percutaneous Coronary Intervention: The Role of Vascular Access Site

**DOI:** 10.3390/jcm13082367

**Published:** 2024-04-18

**Authors:** Stefano Rigattieri, Ernesto Cristiano, Federica Tempestini, Luca Pittorino, Vincenzo Cesario, Matteo Casenghi, Francesca Giovannelli, Antonella Tommasino, Emanuele Barbato, Andrea Berni

**Affiliations:** 1 Cardiology Division, Sant’Andrea University Hospital, Via di Grottarossa 1035, 00189 Rome, Italy; cristianoernesto@gmail.com (E.C.); federicatempestini95@gmail.com (F.T.); luca.pittorino@gmail.com (L.P.); vicesario91@gmail.com (V.C.); matcasenghi@hotmail.it (M.C.); francy_giovannelli@yahoo.it (F.G.); antonellatommasino@gmail.com (A.T.); emanuele.barbato@uniroma1.it (E.B.); andrea.berni@uniroma1.it (A.B.); 2 Department of Electrophysiology, Humanitas Gavazzeni, 24125 Bergamo, Italy; 3 Department of Clinical and Molecular Medicine, Sapienza University of Rome, 00185 Rome, Italy

**Keywords:** STEMI, NSTEMI, multivessel disease

## Abstract

**Background:** in patients undergoing percutaneous coronary interventions (PCI), radial access should be favoured over femoral access as it reduces the risk of vascular complications and bleeding. Furthermore, a preventive role of radial access in the occurrence of acute kidney injury (AKI), mainly mediated by the reduction of bleeding and cholesterol crystal embolization into renal circulation, has been investigated in several studies, yielding conflicting results. **Methods:** we designed a retrospective study to appraise the effect of the use of a vascular access site on the occurrence of AKI in a cohort of 633 patients with acute myocardial infarction treated by PCI at our centre from 2018 to 2020. **Results:** after propensity score adjustment, radial access was associated with a reduced, albeit statistically not significant, incidence of AKI (14.7% vs. 21.0%; *p* = 0.06) and major bleeding (12.5% vs. 18.7%; *p* = 0.04) as compared to femoral access. At multivariate analysis, femoral access was an independent predictor of AKI, together with in-hospital occurrence of BARC 3–5 bleeding, Killip class >1 at presentation, female gender, baseline eGFR <60 mL/min, and baseline haemoglobin <12 g/dL. **Conclusions:** although limited by the observational design, our study supports the hypothesis that radial access may exert a protective role on the occurrence of AKI in patients with acute myocardial infarction undergoing PCI.

## 1. Introduction

Percutaneous coronary interventions (PCI) are associated with a risk of acute kidney injury (AKI), which is mainly driven by the administration of contrast medium [1]. Indeed, iodinated contrast can cause AKI through direct toxicity and increased release of renal vasoconstrictors leading to hemodynamic changes in the renal medulla; in fact, iodinated contrast represents the third leading cause of AKI in hospitalized patients [2,3,4]. The risk of AKI is higher and more clinically relevant in patients with acute coronary syndromes who undergo PCI in an emergent/urgent setting, because these patients cannot be adequately hydrated and often present with a variable degree of left ventricle systolic dysfunction, which has been shown to be a predictor of acute cardio–renal syndrome in ST elevation myocardial infarction [5,6,7]. Use of the radial approach has been consistently shown to be associated with a reduction in vascular complications and bleeding as compared to the femoral approach [8,9]. Furthermore, it has been hypothesized that the radial approach could also lead to a reduced incidence of AKI through reductions in bleeding and, possibly, less cholesterol crystal embolization in renal arteries, as guidewires and catheters do not cross the abdominal aorta [10]. Thus far, several observational studies [11,12,13,14,15,16,17,18,19,20,21,22] and two randomized trials [23,24] have addressed this issue, with conflicting results (Appendix A). In the present study, we compared in-hospital AKI and bleeding incidence according to vascular access site (radial versus femoral) in a cohort of 633 patients with acute myocardial infarction (AMI) undergoing PCI.

## 2. Materials and Methods

### 2.1. Study Design and Population

This study is a post-hoc analysis of a retrospective, single-centre registry aimed to assess the prognostic role of lipoprotein(a) at presentation in patients with AMI undergoing PCI. The design and results of this registry, which was approved by the Ethics Committee of Sapienza University, Rome, Italy, have been already published [25]. Briefly, out of a total population of 889 patients with AMI (both ST elevation and non-ST elevation) undergoing PCI from 2018 to 2020, we selected a cohort of 633 patients for whom creatinine levels before, 24 h after, and 48 h after the procedure were available. AMI was diagnosed in the presence of the rise and fall of high sensitivity cardiac troponin I values with at least one value above the 99th upper reference limit, together with symptoms of myocardial ischemia, new ischemic ECG changes, or echocardiographic evidence of new regional wall motion anomalies. The primary study outcome was the incidence of AKI according to the KDIGO definition (increase in serum creatinine by ≥0.3 mg/dL within 48 h) [26]; the secondary outcome was the incidence of in-hospital BARC 3–5 bleeding according to the Bleeding Academic Research Consortium definition [27]. Patients without troponin increase at presentation, who were treated conservatively or with bypass surgery after coronary angiography, or who needed transfemoral aortic counter pulsation were excluded. Clinical, laboratory, and angiographic data were collected in a dedicated database. The study was approved by the institutional review board of Sapienza University, Rome, Italy.

### 2.2. Statistical Analysis

Continuous variables that were normally distributed were reported as mean ± standard deviation (SD) and were compared using a Student’s *t*-test; continuous variables not normally distributed were reported as median and interquartile range [IQR] and were compared using the Mann–Whitney U test; categorical variables were reported as counts (percentage) and were compared using a chi-squared test. Before outcome analysis, multiple imputation chain equation (MICE) with random forest (RF) and classification and regression tree (CART) were used for imputation in case of missing data. After outcome analysis, results were pooled using Rubin’s rule for variance correction. Outcome comparison analysis was made using inverse probability of treatment weighting (IPTW) and propensity score matching (PSM) to control the differences in baseline characteristics of patients receiving radial or femoral access, rebalancing selection bias. A propensity score for each patient was calculated using covariate balancing propensity score (CBPS) that offered the best common support. The PS estimation included the following variables deemed to possibly affect vascular access choice: age, gender, history of smoking, arterial hypertension, chronic heart failure, NYHA class >1, diabetes mellitus, dyslipidaemia, family history of coronary artery disease, peripheral artery disease, history of myocardial infarction, known vascular disease in any district, body mass index (BMI), body surface area (BSA), type of MI at presentation (ST or non ST-segment elevation), Killip class at presentation >1, baseline haemoglobin, and baseline creatinine. PSM was performed using the 1:1 nearest neighbour matching method and a calliper width of 0.25 standard deviations chosen after optimal matching and assessment of balance of covariates. For IPTW analysis, weights were calculated as the inverse of propensity score. By applicating weights to the study population, a “pseudopopulation” was created in which confounders were balanced across groups [28]. Similarly, survey-weighted generalized logistic models were adopted. Univariable and multivariable logistic regression models were used to estimate the odds ratios (ORs) and 95% confidence intervals (CIs) of the independent association between arterial access and the occurrence of the primary outcome. Finally, sensitivity analysis was performed using the Rosenbaum sensitivity test for PSM [29]. The statistical significance level was set at two-tailed *p* < 0.05. Statistical analyses were performed with R software version 4.3.0 (R Foundation for Statistical Computing).

## 3. Results

**Figure 1:**
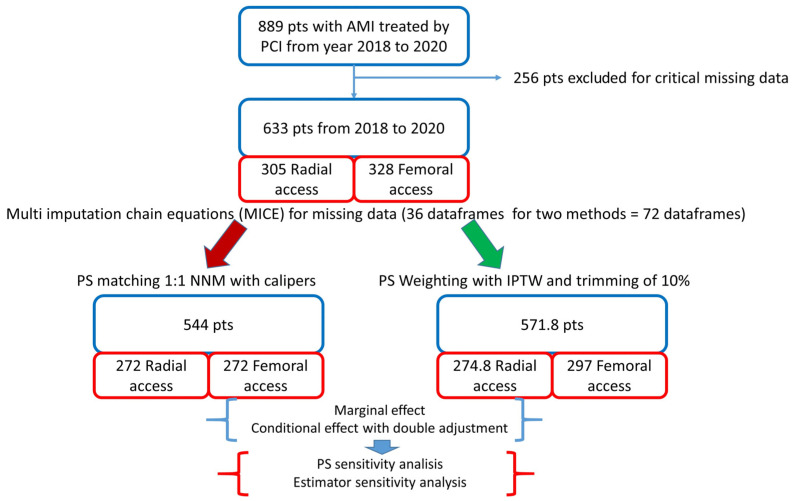
Study design. AMI: acute myocardial infarction; PCI: percutaneous coronary intervention; PS: propensity score; NNM: nearest neighbour matching; IPTW: inverse probability of treatment weighting. In the bottom right panel, the “pseudopopulation”, obtained by weighting each individual by the inverse probability of receiving his/her actual treatment, is shown.

The design and flowchart of the study are described in Figure 1.

Out of 633 patients included, 305 received radial access and 328 femoral access. Patients in the femoral group were older and presented a higher prevalence of female gender, previous MI and ST elevation, and MI at presentation; they also had lower estimated glomerular filtration rate (eGFR), haemoglobin, and haematocrit values as compared to patients in the radial group (Table 1).

Procedural characteristics were similar between the groups, except for a higher number of diseased vessels in the femoral group (Table 2).

**Figure 2:**
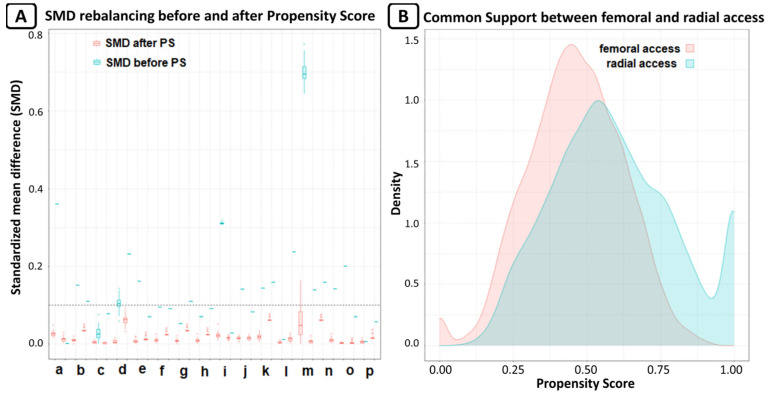
(**A**) Rebalance of variable expressed as standardized mean differences (SMD); SMD lower than 0.1 was considered as good rebalancing. For every variable, there are two box plots; the first for original data, and the second for after MICE estimation for missing data. The variables are: a. age, b. arterial hypertension, c. BMI, d. basal creatinine, e. dyslipidaemia, f. diabetes mellitus, g. familiarity for CAD, h. smoke, i. basal Hb, j. Killip class, k. NYHA class, l. PAD, m. previous cardiovascular event, n. previous HF, o. sex, and p. STEMI. (**B**) Common support of propensity score between radial and femoral access; the graph shows the good overlap between the two groups.

A vascular closure device (Femoseal, Terumo Corporation, Tokyo, Japan) was implanted in 89.6% of patients in the femoral group. Glicoprotein IIb/IIIa inhibitors were used in 5.9% of patients in the radial group and in 7.1% of patients in femoral group (*p* = 0.55). After PSM, we obtained 2 groups of patients treated via radial or femoral access, each consisting of 272 patients. The rebalancing of the variables is shown in Figure 2.

**Figure 3:**
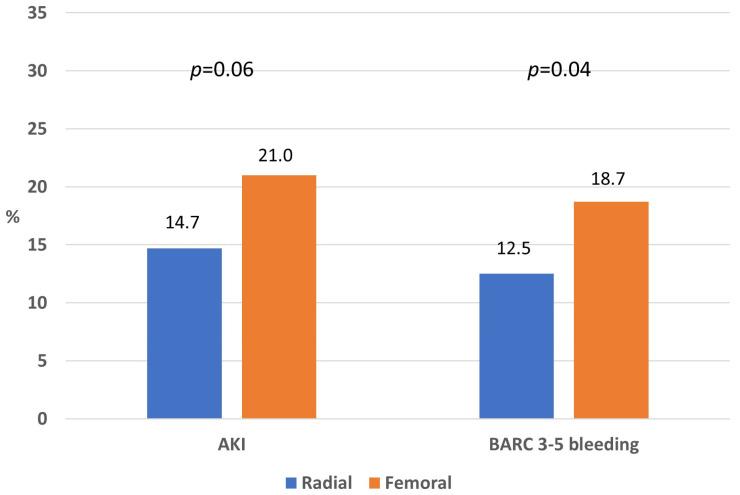
Rate of acute kidney injury and major bleeding in the propensity-matched cohort according to vascular access.

In the PSM cohort, the incidence of AKI was 14.7% in the radial and 21.0% in the femoral group (*p* = 0.06), whereas the incidence of BARC 3–5 bleedings was 12.5% in the radial and 18.7% in the femoral group (*p* = 0.04; Figure 3).

**Figure 4:**
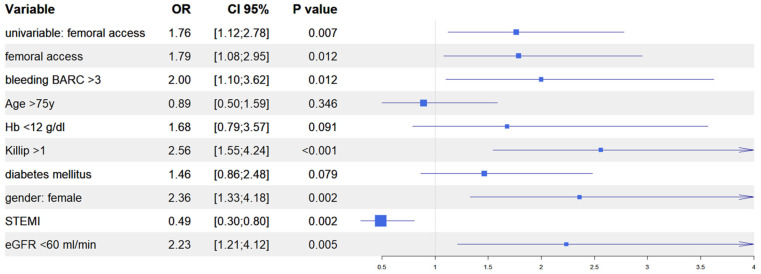
Propensity score weighting: multivariable logistic regression.

**Figure 5:**
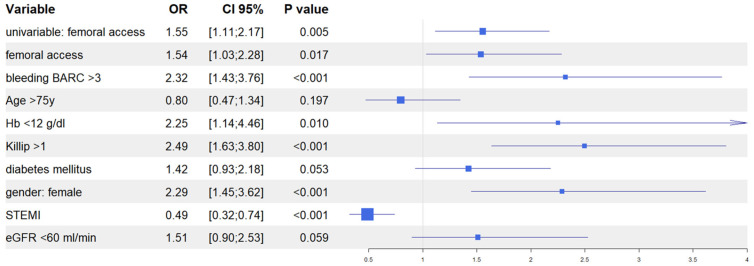
Propensity score matching: multivariable logistic regression.

After univariate analysis (Table S1), we performed multivariate analyses in both the IPTW and in the PSM populations to evaluate the predictive role of vascular access site in the occurrence of AKI. In the IPTW model, femoral access was an independent predictor of AKI together, with in-hospital occurrence of BARC 3–5 bleeding, Killip class >1 at presentation, female gender, and baseline eGFR <60 mL/min; similarly, in the PSM model, femoral access was an independent predictor of AKI, together with in-hospital occurrence of BARC 3–5 bleedings, Killip class >1 at presentation, female gender, and baseline haemoglobin <12 g/dL. Interestingly, in both models, the diagnosis of ST elevation MI at presentation was the only variable that predicted a reduced risk of AKI (Figure 4 and Figure 5). Finally, the Rosenbaum sensitivity test for PSM balance showed that, hypothesizing the presence of an unmeasured covariate, the latter would have affected the result of PSM only if its prevalence would have been up to four times higher in a group as compared to the other, suggesting that the presence of a strong unobserved bias was unlikely.

## 4. Discussion

In this study we observed that radial access, as compared with femoral access, was associated with a reduced, albeit statistically not significant, incidence of AKI in a population of patients with AMI undergoing PCI. There are several pathophysiological mechanisms that could theoretically explain this finding. First, radial access, due to intrinsic anatomic reasons, is associated with a reduced incidence of access-related bleeding which, in turn, may contribute to AKI through hypovolemia, hypotension, and eventually the need for blood transfusions, which represent themselves as risk factors for AKI [30]. Second, the embolization of cholesterol crystals in renal circulation is a well-known, yet overlooked cause of AKI in clinical practice [31]; in this regard, it has been hypothesized that the passage of guidewires and catheters in the abdominal aorta when using femoral access may lead to scraping of atherosclerotic plaques and subsequent embolization which, on the contrary, would be avoided by radial access [32]. The impact of vascular access site on AKI after percutaneous coronary procedures has been the object of several investigations in recent years, but these investigations have produced inconsistent results. A possible protective role of radial access was first described in a large prospective database conducted in British Columbia; this study showed that femoral access was associated with an increased risk of chronic kidney disease onset 6 month after a coronary angiography or PCI as compared with radial access [33]. Similar findings were observed in several observational registries focusing on AKI, whereas other registries showed heterogeneous results (Table 1); indeed, some studies showed a reduced incidence of AKI with radial as compared to femoral access, whereas other studies showed a similar incidence across both access routes. Interestingly, an increased risk of AKI with radial as compared with femoral access was not observed in any study, although procedures performed via radial access may be technically more challenging and associated with suboptimal guiding catheter back up, increased radiation exposure, procedural time and, therefore, contrast volume dose, especially when performed by inexperienced operators. When looking at randomized studies, the AKI-MATRIX showed a reduced incidence of AKI with radial as compared with femoral access (OR 0.87; 95% CI 0.77–0.98); interestingly, the benefits of radial access in this study seemed to be mostly related to the reduction of bleeding, since radial access was no longer an independent predictor when bleeding was incorporated in the multivariate model [23]. Differently, a post-hoc analysis of the SAFARI-STEMI trial showed that radial access was not associated with a reduced incidence of AKI (RR 0.90; 95% CI 0.72–1.13), and it was not an independent predictor of AKI in multivariate modelling, irrespective of the inclusion of bleedings [24]; however, the fact that in the main trial, no significant difference was observed in the rate of bleeding between the two access routes reinforces the notion that the main mechanism through which radial access may prevent AKI is through the reduction of bleeding events. This notwithstanding, both in our study and in other studies, the protective effect of radial access has been found to be independent of its beneficial effect on bleeding; in this regard, the reduced rate of cholesterol embolization in renal circulation associated with radial access could represent a further protective mechanism. Cholesterol crystal embolism represents an elusive and overlooked clinical entity; indeed, a definite diagnosis can only be made with cutaneous biopsy, whereas a probable diagnosis relies on cutaneous signs (livedo reticularis, blue toe syndrome, and digital gangrene) or laboratory findings (renal impairment or eosinophilia). Cholesterol crystal embolism associated with PCI, although infrequent, is associated with long-term death and need for chronic haemodialysis, and is more prevalent with femoral as compared to radial access, showing a tendency to decline over years when transitioning from femoral to radial access [34]. An interesting finding of our study is that the diagnosis of STEMI was the only protective clinical variable against AKI, both in the IPTW and the PSM multivariate models. A possible explanation for this is that, similarly to what has been observed in previous studies [35], in our population, patients with STEMI were younger and had a lower prevalence of cardiovascular risk factors, anaemia, heart failure and peripheral artery disease as compared with patients with NSTEMI, thus representing a population at reduced risk of AKI.

Our study presents several limitations. First, it is a post-hoc analysis of a single-centre, retrospective registry; therefore, despite statistical adjustment for possible clinical and procedural confounders, a selection bias favouring radial access cannot be excluded. Moreover, the single-centre design further limits the generalizability of the results. Second, the amount of contrast medium given to patients was not collected; it is worth noting, however, that a significant difference in this regard between radial and femoral access has never been observed either in randomized trials or in propensity-matched studies (Appendix A). Third, data on hydration protocols before and after PCI were not collected.

These limitations notwithstanding, our data support the notion that radial access is associated with a reduced rate of bleeding events and AKI in patients with AMI undergoing PCI as compared with femoral access, even with a low rate of Gp IIb/IIIa inhibitors and a high rate of femoral vascular closure device use, both of which reflect contemporary clinical practices. In this regard, vascular closure devices have been shown to be inferior to radial access in the prevention of bleeding and vascular complications in a meta-analysis of randomized and observational studies [36]. Given the negative prognostic role of bleeding events and AKI in patients with acute coronary syndromes [37,38], the adoption of radial access, together with the prevention of hypovolemia, the avoidance of nephrotoxic agents, and the minimization of contrast dose represent effective strategies that should be implemented whenever possible in the management of these patients.

## Figures and Tables

**Table 1:** Clinical characteristics of the study population.

	Radial Access	Femoral Access	*p* Value
n	305	328	
Age (years)	64 ± 11	67 ± 12	<0.001
Female gender (n, %)	61 (20.0)	89 (27.1)	0.044
BMI	27 ± 4	27 ± 4	0.839
History of smoking (n, %)	210 (69.0)	212 (64.6)	0.346
Hypertension (n, %)	215 (70.5)	248 (75.6)	0.118
Heart failure (n, %)	27 (8.9)	42 (12.8)	0.125
Diabetes (n, %)	79 (25.9)	95 (28.9)	0.412
Dyslipidemia (n, %)	190 (62.3)	222 (67.7)	0.164
Peripheral artery disease (n, %)	28 (9.2)	28 (8.5)	0.903
Previous stroke (n, %)	12 (3.9)	17 (5.2)	0.564
Previous myocardial infarction (n, %)	38 (12.5)	65 (19.8)	0.015
Previous CABG (n, %)	10 (3.3)	16 (4.9)	0.408
STEMI (n, %)	153 (50.2)	198 (60.4)	0.008
Killip class >1 (n, %)	61 (20.0)	85 (25.9)	0.069
eGFR (mL/min)	90 ± 31	80 ± 34	<0.001
Hemoglobin (g/dL)	15 ± 2	14 ± 2	<0.001
Hematocrit (%)	44 ± 5	42 ± 6	0.001

BMI: body mass index; CABG: coronary artery bypass grafting; STEMI: ST segment elevation myocardial infarction; eGFR: estimated glomerular filtration rate.

**Table 2:** Procedural characteristics of the study population.

	Radial Access	Femoral Access	*p* Value
n	305	328	
Diseased vessels:			
- LAD (n, %)	232 (76.1)	265 (80.8)	0.112
- Circ (n, %)	154 (50.5)	176 (53.7)	0.402
- RCA (n, %)	140 (45.9)	191 (58.2)	0.002
- Left main (n, %)	21 (6.9)	25 (7.6)	0.814
- Bypass (n, %)	10 (3.3)	11 (3.3)	1.000
n of diseased vessels	2 [1, 2]	2 [1, 3]	0.001
n of implanted stent	2 [1, 3]	2 [1, 3]	0.257
n of treated vessels	1 [1, 2]	1 [1, 2]	0.096
Total stent length (mm)	37 [23, 58]	38 [24, 67]	0.122
Vascular closure device (n, %)	0 (0.0)	294 (89.6)	<0.001

LAD: left anterior descending coronary artery; Circ: circumflex coronary artery; RCA: right coronary artery.

## Data Availability

All data not included in the manuscript are available via contacting the corresponding author in accordance with local legal regulations.

## Supplementary Materials

The following supporting information can be downloaded at: https://www.mdpi.com/article/10.3390/jcm13082367/s1, Table S1: Univariate predictors of AKI.

## Appendix A

Studies investigating the impact of vascular access (radial or femoral) on the occurrence of acute kidney injury following percutaneous coronary interventions.

**Table:**

Randomized Controlled Trials								
Study	Year	Design	Clinical Setting	N	AKI Definition	AKI	Contrast Volume	Bleedings
Andò et al. [23]	2017	Multicentre, prospective randomized controlled trial (pre-specified analysis)	Angio and/or PCI in ACS	8210	Absolute (>0.5 mg/dL) or relative (>25%) increase from baseline creatinine KDIGO	R < F R = F	R = F	R < F
Marbach et al. [24]	2021	Multicentre, prospective randomized controlled trial (post hoc analysis)	Primary PCI	2285	Absolute (>0.5 mg/dL) or relative (>25%) increase from baseline creatinine KDIGO	R = F	R = F	R = F
	Observational studies with propensity matching							
Study	Year	Design	Clinical setting	N		AKI	Contrast volume	Bleedings
Cortese et al. [11]	2014	Observational, retrospective, multicentre, propensity matched	Primary PCI	450	Absolute (>0.5 mg/dL) or relative (>25%) increase from baseline creatinine	R < F	R = F	R < F
Kooiman et al. [12]	2014	Observational, retrospective, multicentre, propensity matched	PCI	17,716	Absolute (>0.5 mg/dL) or relative (>25%) increase from baseline creatinine	R < F	NR	R < F
Kolte et al. [13]	2016	Observational, retrospective, multicentre, propensity-matched	Primary PCI	508	Absolute (>0.5 mg/dL) or relative (>25%) increase from baseline creatinine	R = F	R = F	NR
Steinvil et al. [14]	2017	Observational, single-centre, propensity matched	PCI	1072	increase of ≥0.3 mg/dL in sCr or 1.5- to 2-fold increase in maximal sCr compared with baseline sCr at admission	R < F	R = F	R = F
Kanic et al. [15]	2019	Observational, retrospective, single-centre, propensity matched	PCI for AMI	2098	Absolute increase of creatinine ≥0.3 or 0.5 in the first 48 h after PCI KDIGO	R = F	NR	NR
	Other observational studies							
Study	Year	Design	Clinical setting	N		AKI	Contrast volume	Bleedings
Damluji et al. [16]	2014	Observational, retrospective, single-centre, propensity-adjusted	Angio and/or PCI	1637	AKI was defined as a rise in serum creatinine N0.5 (mg/dL) or 50% from the baseline value within 72 h post cardiac catheterization or PCI.	R = F	R > F (unadjusted)	NR
Pancholy et al. [17]	2017	Observational, retrospective, single-centre propensity adjusted	PCI	7529	>0.5 mg/dL or >25% increase in serum creatinine level at 48 to 72 h after the PCI procedure compared with baseline	R < F	R < F (unadj.)	R < F (unadj.)
Feldkamp et al. [18]	2017	Observational, retrospective, single-centre	Angio and/or PCI	2937	KDIGO	R < F	R = F (unadj.)	NR
Barbieri et al. [19]	2018	Observational, prospective, single-centre, propensity adjusted	Angio and/or PCI	4199	>0.5 mg/dL or 25% of baseline levels, within 24–48 h after contrast exposure	R = F	R < F (unadj.)	NR
Stephan et al. [20]	2021	Observational Retrospective Single-centre	Angio and/or PCI in CABG	527	KDIGO	R = F	R < F (unadj.)	R = F (unadj.)
Carande et al. [21]	2022	Observational Retrospective Single-centre	PCI in ACS	2747	Increase in serum creatinine by ≥26 µmol/L within 48 h or increase ≥1.5 times	R < F	NR	NR
Kietrsunthorn et al. [22]	2023	Observational, retrospective, multicentre	PCI	14,077	absolute increase of ≥0.3 mg/dL or a relative increase of 50% in serum creatinine or a new requirement for dialysis following PCI	R < F	NR	NR

AKI: acute kidney injury; R: radial access; F: femoral access; PCI: percutaneous coronary intervention; ACS: acute coronary syndrome; AMI: acute myocardial infarction; CABG: coronary artery bypass grafting.

## References

[1] McCullough P.A., Choi J.P., Feghali G.A., Schussler J.M., Stoler R.M., Vallabahn R.C., Mehta A. (2016). Contrast-Induced Acute Kidney Injury. J. Am. Coll. Cardiol..

[2] Bui K.L., Horner J.D., Herts B.R., Einstein D.M. (2007). Intravenous iodinated contrast agents: Risks and problematic situations. Cleve Clin. J. Med..

[3] Finn W.F. (2006). The clinical and renal consequences of contrast-induced nephropathy. Nephrol. Dial. Transplant..

[4] Tumlin J., Stacul F., Adam A., Becker C.R., Davidson C., Lameire N., McCullough P.A. (2006). Pathophysiology of contrast-induced nephropathy. Am. J. Cardiol..

[5] Giacoppo D., Madhavan M.V., Baber U., Warren J., Bansilal S., Witzenbichler B., Dangas G.D., Kirtane A.J., Xu K., Kornowski R. (2015). Impact of Contrast-Induced Acute Kidney Injury after Percutaneous Coronary Intervention on Short- and Long-Term Outcomes: Pooled Analysis from the HORIZONS-AMI and ACUITY Trials. Circ. Cardiovasc. Interv..

[6] Di Lullo L., Bellasi A., Russo D., Cozzolino M., Ronco C. (2017). Cardiorenal acute kidney injury: Epidemiology, presentation, causes, pathophysiology and treatment. Int. J. Cardiol..

[7] Khoury S., Steinvil A., Gal-Oz A., Margolis G., Hochstatd A., Topilsky Y., Keren G., Shacham Y. (2018). Association between central venous pressure as assessed by echocardiography, left ventricular function and acute cardio-renal syndrome in patients with ST segment elevation myocardial infarction. Clin. Res. Cardiol..

[8] Agostoni P., Biondi-Zoccai G.G., De Benedictis M., Rigattieri S., Turri M., Anselmi M., Vassanelli C., Zardini P., Louvard Y., Hamon M. (2004). Radial versus femoral approach for percutaneous coronary diagnostic and interventional procedures: Systematic overview and meta-analysis of randomized trials. J. Am. Coll. Cardiol..

[9] Ferrante G., Rao S.V., Jüni P., Da Costa B.R., Reimers B., Condorelli G., Anzuini A., Jolly S.S., Bertrand O.F., Krucoff M.W. (2016). Radial Versus Femoral Access for Coronary Interventions across the Entire Spectrum of Patients with Coronary Artery Disease: A Meta-Analysis of Randomized Trials. JACC Cardiovasc. Interv..

[10] Andò G., Cortese B., Frigoli E., Gagnor A., Garducci S., Briguori C., Rubartelli P., Calabrò P., Valgimigli M. (2015). Acute kidney injury after percutaneous coronary intervention: Rationale of the AKI-MATRIX (acute kidney injury-minimizing adverse hemorrhagic events by TRansradial access site and systemic implementation of angioX) sub-study. Catheter. Cardiovasc. Interv..

[11] Cortese B., Sciahbasi A., Sebik R., Rigattieri S., Alonzo A., Silva-Orrego P., Belloni F., Seregni R.G., Giovannelli F., Tespili M. (2014). Comparison of risk of acute kidney injury after primary percutaneous coronary interventions with the transradial approach versus the transfemoral approach (from the PRIPITENA urban registry). Am. J. Cardiol..

[12] Kooiman J., Seth M., Dixon S., Wohns D., LaLonde T., Rao S.V., Gurm H.S. (2014). Risk of acute kidney injury after percutaneous coronary interventions using radial versus femoral vascular access: Insights from the Blue Cross Blue Shield of Michigan Cardiovascular Consortium. Circ. Cardiovasc. Interv..

[13] Kolte D., Spence N., Puthawala M., Hyder O., Tuohy C.P., Davidson C.B., Sheldon M.W., Laskey W.K., Abbott J.D. (2016). Association of radial versus femoral access with contrast-induced acute kidney injury in patients undergoing primary percutaneous coronary intervention for ST-elevation myocardial infarction. Cardiovasc. Revasc. Med..

[14] Steinvil A., Garcia-Garcia H.M., Rogers T., Koifman E., Buchanan K., Alraies M.C., Torguson R., Pichard A.D., Satler L.F., Ben-Dor I. (2017). Comparison of Propensity Score-Matched Analysis of Acute Kidney Injury after Percutaneous Coronary Intervention with Transradial versus Transfemoral Approaches. Am. J. Cardiol..

[15] Kanic V., Kompara G., Šuran D., Tapajner A., Naji F.H., Sinkovic A. (2019). Acute kidney injury in patients with myocardial infarction undergoing percutaneous coronary intervention using radial versus femoral access. BMC Nephrol..

[16] Damluji A., Cohen M.G., Smairat R., Steckbeck R., Moscucci M., Gilchrist I.C. (2014). The incidence of acute kidney injury after cardiac catheterization or PCI: A comparison of radial vs. femoral approach. Int. J. Cardiol..

[17] Pancholy S.B., Patel G.A., Patel N.R., Patel D.D., Patel P., Pandya S.M., Verma A.A., Shah S.C., Mamas M.A., Patel T.M. (2021). Trends, Outcomes, and Predictive Score for Emergency Coronary Artery Bypass Graft Surgery after Elective Percutaneous Coronary Intervention (from a Nationwide Dataset). Am. J. Cardiol..

[18] Feldkamp T., Luedemann M., Spehlmann M.E., Freitag-Wolf S., Gaensbacher J., Schulte K., Bajrovic A., Hinzmann D., Hippe H.-J., Kunzendorf U. (2018). Radial access protects from contrast media induced nephropathy after cardiac catheterization procedures. Clin. Res. Cardiol..

[19] Barbieri L., Verdoia M., Suryapranata H., De Luca G. (2019). Impact of vascular access on the development of contrast induced nephropathy in patients undergoing coronary angiography and/or percutaneous coronary intervention. Int. J. Cardiol..

[20] Stephan T., Felbel D., Rattka M., Rottbauer W., Markovic S. (2022). Impact of Radial Access on Contrast-Induced Acute Kidney Injury in Patients with Coronary Artery Bypass Grafts. Cardiovasc. Revascularization Med..

[21] Carande E.J., Brown K., Jackson D., Maskell N., Kouzaris L., Greene G., Mikhail A., Obaid D.R. (2022). Acute Kidney Injury Following Percutaneous Coronary Intervention for Acute Coronary Syndrome: Incidence, Aetiology, Risk Factors and Outcomes. Angiology.

[22] Kietrsunthorn P.S., Locklear T.M., Fonner C.E., Berzingi C.O., Foerst J.R., Mirza M.A., Sane D.C., Williams E., Shor R.A., Dehmer G.J. (2023). Association of Radial Artery Access with Reduced Incidence of Acute Kidney Injury. J. Interv. Cardiol..

[23] Andò G., Cortese B., Russo F., Rothenbühler M., Frigoli E., Gargiulo G., Briguori C., Vranckx P., Leonardi S., Guiducci V. (2017). Acute Kidney Injury after Radial or Femoral Access for Invasive Acute Coronary Syndrome Management: AKI-MATRIX. J. Am. Coll. Cardiol..

[24] Marbach J.A., Wells G., Di Santo P., So D., Chong A.-Y., Russo J., Labinaz M., Dick A., Froeschl M., Glover C. (2021). Acute kidney injury after radial or femoral artery access in ST-segment elevation myocardial infarction: AKI-SAFARI. Am. Hear. J..

[25] Rigattieri S., Cristiano E., Tempestini F., Monaco M.L., Cava F., Bongiovanni M., Tifi P., Berni A., Volpe M. (2023). Lipoprotein(a) and the risk of recurrent events in patients with acute myocardial infarction treated by percutaneous coronary intervention. Minerva Cardioangiol..

[26] KDIGO Clinical Practice Guideline for Acute Kidney Injury. http://www.kidney-international.org.

[27] Mehran R., Rao S.V., Bhatt L.D., Gibson C.M., Caixeta A., Eikelboom J., Kaul S., Wiviott S.D., Menon V., Nikolsky E. (2011). Special Report Standardized Bleeding Definitions for Cardiovascular Clinical Trials A Consensus Report from the Bleeding Academic Research Consortium. Circulation.

[28] Chesnaye N.C., Stel V.S., Tripepi G., Dekker F.W., Fu E.L., Zoccali C., Jager K.J. (2021). An introduction to inverse probability of treatment weighting in observational research. Clin. Kidney J..

[29] Liu W., Kuramoto S.J., Stuart E.A. (2013). An introduction to sensitivity analysis for unobserved confounding in nonexperimental prevention research. Prev Sci..

[30] Karrowni W., Vora A.N., Dai D., Wojdyla D., Dakik H., Rao S.V. (2016). Blood Transfusion and the Risk of Acute Kidney Injury among Patients with Acute Coronary Syndrome Undergoing Percutaneous Coronary Intervention. Circ. Cardiovasc. Interv..

[31] Scolari F., Ravani P. (2010). Atheroembolic renal disease. Lancet.

[32] Keeley E., Grines C.L. (1998). Scraping of aortic debris by coronary guiding catheters: A prospective evaluation of 1000 cases. J. Am. Coll. Cardiol..

[33] Vuurmans T., Byrne J., Fretz E., Janssen C., Hilton J.D., Klinke W.P., Djurdjev O., Levin A. (2010). Chronic kidney injury in patients after cardiac catheterisation or percutaneous coronary intervention: A comparison of radial and femoral approaches (from the British Columbia Cardiac and Renal Registries). Heart.

[34] Takahashi K., Omuro A., Ohya M., Kubo S., Tada T., Tanaka H., Fuku Y., Kadota K. (2022). Incidence, Risk Factors, and Prognosis of Cholesterol Crystal Embolism Because of Percutaneous Coronary Intervention. Am. J. Cardiol..

[35] Montalescot G., Dallongeville J., Van Belle E., Rouanet S., Baulac C., Degrandsart A., Vicaut E. (2007). STEMI and NSTEMI: Are they so different? 1 year outcomes in acute myocardial infarction as defined by the ESC/ACC definition (the OPERA registry). Eur. Hear. J..

[36] Rigattieri S., Sciahbasi A., Ratib K., Alonzo A., Cox N., Chodór P., Berni A., Fedele S., Pugliese F.R., Cooper C.J. (2016). Comparison between Radial Approach and Femoral Approach with Vascular Closure Devices on the Occurrence of Access-Site Complications and Periprocedural Bleeding after Percutaneous Coronary Procedures: A Systematic Review and Meta-Analysis. J. Invasive Cardiol..

[37] Laudani C., Capodanno D., Angiolillo D.J. (2023). Bleeding in acute coronary syndrome: From definitions, incidence, and prognosis to prevention and management. Expert Opin. Drug Saf..

[38] Marenzi G., Cosentino N., Bartorelli A.L. (2015). Acute kidney injury in patients with acute coronary syndromes. Heart.

