# Supplementary material for: Acute Kidney Injury in Patients with Acute Myocardial Infarction Undergoing Percutaneous Coronary Intervention: The Role of Vascular Access Site
Source: J Clin Med. 2024 Apr 18;13(8):2367. doi: 10.3390/jcm13082367 (PMC11051292; doi:10.3390/jcm13082367)
Supplement: Supplementary file 1 [file jcm-13-02367-s001.zip › jcm-2905599-supplementary.pdf]

Table S1. Univariate predictors of AKI.

	coefficient	SE	lower	upper	p_value
Female gender	1.12	0.23	0.6692	1.5708	<0.001
Hypertension	0.23	0.25	-0.26	0.72	0.361
Diabetes	0.55	0.23	0.0992	1.0008	0.017
age >75	0.83	0.25	0.34	1.32	<0.001
STEMI	-0.64	0.21	-1.0516	-0.2284	0.002
dyslipidemia	-0.1	0.22	-0.5312	0.3312	0.668
NYHA >1	0.66	0.32	0.0328	1.2872	0.039
Previous MI	0.34	0.28	-0.2088	0.8888	0.225
Family history	-0.08	0.23	-0.5308	0.3708	0.725
Killip >1	1.09	0.24	0.6196	1.5604	<0.001
eGFR<60	1.35	0.25	0.86	1.84	<0.001
Hb < 12 g/dl	1.41	0.31	0.8024	2.0176	<0.001
BARC 3-5	0.6	0.26	0.0904	1.1096	0.021
anemia	0.78	0.27	0.2508	1.3092	0.003
